# Application of Linear Mixed-Effects Models in Human Neuroscience Research: A Comparison with Pearson Correlation in Two Auditory Electrophysiology Studies

**DOI:** 10.3390/brainsci7030026

**Published:** 2017-02-27

**Authors:** Tess K. Koerner, Yang Zhang

**Affiliations:** 1 Department of Speech-Language-Hearing Sciences, University of Minnesota, Minneapolis, MN 55455, USA; koern030@umn.edu; 2 Center for Neurobehavioral Development, University of Minnesota, Minneapolis, MN 55455, USA; 3 Center for Applied and Translational Sensory Science, University of Minnesota, Minneapolis, MN 55455, USA; 4 Speech-Language-Hearing Center, School of Foreign Languages, Shanghai Jiao Tong University, Shanghai 200240, China

**Keywords:** Pearson correlation, linear mixed-effects regression models, repeated measures, neurophysiology, event-related potential

## Abstract

Neurophysiological studies are often designed to examine relationships between measures from different testing conditions, time points, or analysis techniques within the same group of participants. Appropriate statistical techniques that can take into account repeated measures and multivariate predictor variables are integral and essential to successful data analysis and interpretation. This work implements and compares conventional Pearson correlations and linear mixed-effects (LME) regression models using data from two recently published auditory electrophysiology studies. For the specific research questions in both studies, the Pearson correlation test is inappropriate for determining strengths between the behavioral responses for speech-in-noise recognition and the multiple neurophysiological measures as the neural responses across listening conditions were simply treated as independent measures. In contrast, the LME models allow a systematic approach to incorporate both fixed-effect and random-effect terms to deal with the categorical grouping factor of listening conditions, between-subject baseline differences in the multiple measures, and the correlational structure among the predictor variables. Together, the comparative data demonstrate the advantages as well as the necessity to apply mixed-effects models to properly account for the built-in relationships among the multiple predictor variables, which has important implications for proper statistical modeling and interpretation of human behavior in terms of neural correlates and biomarkers.

## 1. Introduction

Cognitive neuroscience research aims to explore relationships between various neural and behavioral measures to examine the underlying peripheral/central neural mechanisms in various testing conditions and subject populations. For this purpose, the bivariate Pearson correlation analysis is commonly used to examine the strength of the linear relationship between two continuous variables of interest, which can be graphically represented by fitting a least-squares regression line in a scatter plot [1,2]. If the variables do not represent continuous data or if the relationship between the two variables is non-linear, other types of bivariate correlation tests such as Spearman or Point-Biserial correlations can be used. However, when a study involves multivariate data, the conventional correlation method only allows for the examination of one predictor and one outcome variable at a time. Even if the Pearson correlation results are adjusted for multiple comparisons or a simple multiple regression model is applied, the statistical treatment may not take into account the complex relationships and categorical grouping terms that likely exist in the multiple within-subject predictor variables [2]. 

**Figure 1:**
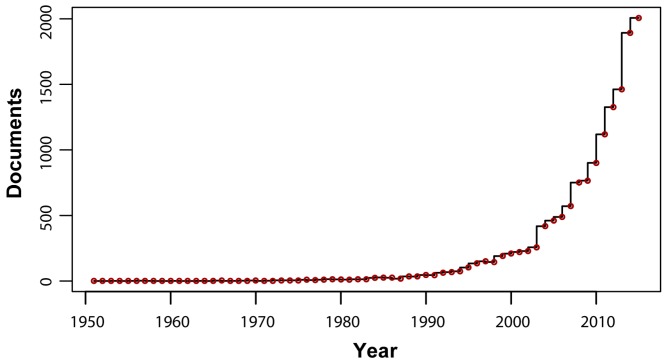
Number of publication documents (including original articles and reviews) from 1951 to 2016 that contain the keyword “linear mixed-effects model”. Literature search was conducted with Elsevier’s Scopus database [53].

In consideration of the violation of the assumed sample independence required of bivariate Pearson correlations and the like, researchers have long argued for the necessity to apply more sophisticated statistical techniques to handle repeated measures from the same subjects [3,4,5]. The use of mixed-effects (or multilevel) models has recently captured attention in longitudinal medical research [6,7,8,9,10,11,12,13,14], behavioral and social sciences research [15,16,17,18,19] (including speech and hearing research [20,21,22,23,24,25,26,27,28,29,30,31,32,33,34,35,36,37,38,39,40,41]), and neurophysiological and neuroimaging research [42,43,44,45,46,47,48,49,50,51,52]. Its increasing popularity is shown in the exponential growth over the last three decades in the number of publications in the scientific literature (Figure 1).

Data analysis using mixed-effects regression models allows for the examination of how multiple variables predict an outcome measure of interest beyond what a simple multiple regression model can handle [2,3,4,5]. In addition to the fixed effects in a conventional multiple regression model, a mixed-effects model includes random effects associated with individual experimental units that have prior distributions. Thus mixed-effects models are able to represent the covariance structure that is inherent in the experimental design. In particular, the linear and generalized linear mixed-effects models (LME or GLME), as implemented in popular software packages such as R, prove to be a powerful tool that allows researchers to examine the effects of several predictor variables (or fixed effects) and their interactions on a particular outcome variable while taking into account grouping factors and the existing covariance structure in the repeated measures data. For instance, adding research participants as a random effect in a LME model allows investigators to resolve the issue of independence among repeated measures by controlling for individual variation among participants. Essentially, the inclusion of subject as a random effect in the model assumes that each participant has a unique intercept, or “baseline”, for each variable. Linear mixed-effects models also allow for an understanding of how changes in an individual predictor variable, among other co-existing variables, impact the outcome measure. These regression coefficients provide more detailed information about relationships among predictors and outcome variables than Pearson correlation coefficients as the Pearson correlation coefficient simply measures the strength of the linear relationship between each selected pair of variables independent of the others. Additionally, driven by the research questions and the nature of the independent and dependent variables, researchers can build and compare LME models differing in complexity to best summarize findings. Many possibilities regarding appropriate types of models, necessary data transformations to achieve linearity for each variable, and the inclusion of interaction terms as well as random slopes or intercepts can be considered. 

Despite the wide acceptance of the LME method and similar approaches for multivariate data analysis, researchers do not necessarily take into account the differences between Pearson correlation and LME models for proper statistical treatment of their data. The current report of side-by-side comparison was propelled by the successive publication of two recent studies from our lab that respectively used conventional Pearson correlations and the more sophisticated linear mixed-effects regression models. In particular, our first study investigated whether noised-induced trial-by-trial changes in cortical oscillatory rhythms in the ongoing auditory electroencephalography (EEG) signal could account for the basic evoked response components in the averaged event-related potential (ERP) waveforms for speech stimuli in quiet and noisy listening conditions [54]. When the first study was submitted, we were not aware of the importance and relevance of the LME approach to the analysis of our data set. Even though the paper went through two rounds of revisions, the two anonymous peer reviewers did not raise any concerns for the use of Pearson correlation in our analysis. Our second study further examined whether the noise-induced changes in trial-by-trial neural phase locking, as measured by inter-trial phase coherence (ITPC) and spectral EEG power, could predict averaged mismatch negativity (MMN) responses for detecting a consonant change and a vowel change and whether the cortical MMN response itself could predict speech perception in noise at both the syllable and sentence levels [54]. In the publication process of the second study, reviewers questioned the validity of the Pearson correlation analysis for the multiple measures for the same speech stimuli from the same group of subjects, which led to a major revision adopting the LME regression analysis. In hindsight, as the trial-by-trial oscillations and the averaged ERPs are different analysis techniques applied to the same EEG signal, it would have been appropriate to choose the LME models to report the statistical results in our first publication. 

As these two previous publications in auditory neuroscience reported only correlation results using one statistical approach, a direct comparison of both the Pearson correlation and LME approaches can be helpful to highlight the differences in the statistical results. Although our examples here are exclusively focused on speech perception research, the informative comparisons of the statistical results are presented as a further development to advocate for proper implementation of statistical modeling and interpretation of multivariate data analysis in future studies of cognitive neuroscience and experimental psychology. 

## 2. Study 1

Koerner and Zhang [54] aimed to determine whether noise-induced changes in trial-by-trial neural synchrony in delta (0.5–4 Hz), theta (4–8 Hz), and alpha (8–12 Hz) frequency bands in response to the syllable /bu/ in quiet and in speech babble background noise at a −3 dB SNR (signal-to-noise ratio) were predictive of variation in the N1–P2 ERPs across participants. 

### 2.1. Statistical Methods

In the published data [54], Pearson correlations were used to examine the strength of linear relationships between ITPC and the N1–P2 amplitude and latency measures pooled across the two listening conditions for each participant and frequency band, resulting in 12 correlations. The reported *p*-values were adjusted for multiple comparisons. Prior to this analysis, scatterplots were used to check the linearity of each pair of continuous variables. Separate repeated measures analysis of variance (ANOVA) were also used to examine the effects of background noise on ITPC and N1–P2 latency and amplitude measures. The ITPC values ranged from 0 to 1, where 1 represents perfect synchronization across trials and 0 represents absolutely no synchronization across trials. Resulting *p*-values were adjusted for multiple comparisons. For the current comparative report, linear mixed-effects models were developed using R [55] and the *nlme* package [56]. Participants were used as a “by-subject” random effect and listening condition (quiet vs. noise) was included as a blocking variable in each linear mixed-effect model. ITPC values at time points associated with the N1 and P2 responses in delta, theta, and alpha frequency bands were included as fixed effects. For each Pearson correlation and linear mixed-effects model, the significance of each variable in predicting behavioral performance was assessed with the significance level at 0.05. 

### 2.2. Results

Koerner and Zhang [54] provided detailed results from repeated measures ANOVAs and the Pearson correlations (see replicated Table 1 for summary of correlation coefficients). The repeated measures ANOVA revealed significant noise-induced delays in N1 (*F*(1, 10) = 53.71, *p* < 0.001) and P2 (*F*(1, 10) = 22.27, *p* < 0.001) latency as well as a significant reduction in N1 amplitude (*F*(1, 10) = 13.85, *p* < 0.01). Additionally, the repeated measures ANOVA revealed significant noise-induced reductions in ITPC for N1 in delta (*F*(1, 10) = 20.68, *p* < 0.01), theta (*F*(1, 10) = 18.51, *p* < 0.01), and alpha (*F*(1, 10) = 23.45, *p* < 0.001) frequency bands as well as for P2 in delta (*F*(1, 10) = 13.27, *p* < 0.01), theta (*F*(1, 10) = 14.86, *p* < 0.01), and alpha (*F*(1, 10) = 14.57, *p* < 0.001) frequency bands.

Results from the Pearson correlation tests showed that ITPC was significantly correlated with N1 latency in delta (*r* = −0.586, *p* < 0.01), theta (*r* = −0.521, *p* < 0.05), and alpha (*r* = −0.510, *p* < 0.05) frequency bands. Similarly, significant correlations were found between ITPC and N1 amplitude in delta (*r* = 0.780, *p* < 0.001), theta (*r* = −0.765, *p* < 0.001), and alpha (*r* = −0.720, *p* < 0.001) frequency bands. Correlational analysis also revealed significant correlations between ITPC and P2 latency in delta (*r* = −0.468, *p* < 0.05), theta (*r* = −0.575, *p* < 0.01), and alpha (*r* = −0.586, *p* < 0.01) frequency bands as well as between ITPC and P2 amplitude in delta (*r* = 0.666, *p* < 0.01), theta (*r* = 0.612, *p* < 0.01), and alpha (*r* = 0.599, *p* < 0.01) frequency bands. 

Results from the linear mixed-effects models showed that ITPC in the delta frequency band was a significant predictor of N1 (*F*(1, 7) = 16.12, *p* < 0.01) and P2 amplitude (*F*(1, 7) = 10.72, *p* < 0.05) across listening conditions. Neural synchrony in the alpha frequency band was a significant predictor of N1 latency (*F*(1, 7) = 12.51, *p* < 0.05) across listening conditions. Potential interaction effects were statistically nonsignificant when examined in a full LME model and were therefore removed from the report. An examination of regression coefficients allows for an interpretation of how each fixed effect is related to the outcome measure of interest. For example, a one-point decrease in ITPC in the delta frequency band is associated with a 1.05 unit increase in the N1 amplitude (see Table 2 for a summary of F-statistics and correlation coefficients (B)). The residual plots from each linear mixed-effects model were normally distributed and did not reveal heteroscedasticity or significant trends. Therefore, it is not expected that generalized linear models would provide better results. 

## 3. Study 2

Koerner et al. [57] aimed to examine whether noise-induced changes in the MMN and spectral power in the theta frequency band in response to a consonant change (/ba/ to /da/) and vowel change (/ba/ to /bu/) in a double-oddball paradigm were predictive of speech perception in noise at the syllable and sentence levels. 

### 3.1. Statistical Methods

For a direct comparison, Pearson correlations were used to examine correlations between the objective MMN (latency, amplitude, and EEG theta power) in response to /da/ and /bu/ and behavioral responses (percent correct phoneme detection, reaction time, and percent correct sentence recognition) pooled across quiet and speech babble noise listening conditions, resulting in 18 correlations. A check of linearity was performed on each pair of continuous variables using scatterplots. Final *p*-values for each correlation coefficient were adjusted to account for multiple comparisons. As reported in Koerner et al. [57], repeated measures ANOVAs were used to examine the effects of background noise on MMN latency, amplitude, and EEG theta power. Linear mixed-effects models were developed to determine whether these objective neural measures were able to predict behavioral performance. Participant was included as a “by-subject” random effect in each linear mixed-effect model while listening condition (quiet vs. noise) and stimulus (/da/ vs. /bu/) were included as blocking (or grouping) variables in each linear mixed-effect model. MMN latency, amplitude, and theta power were added as fixed effects in models with percent correct phoneme detection or reaction time as outcome variables. Similar models were developed to examine whether MMN latency, amplitude, and theta power in response to /da/ or /bu/ were able to predict sentence-level perception using listening condition as a blocking variable. Data transformations for the linear mixed-effects models included re-scaling the MMN latency and behavioral reaction times for phoneme detection as well as log-transforming the percent correct phoneme detection and sentence recognition scores to account for skewness in the data. The significance of each correlation coefficient from the Pearson correlation analysis as well as each fixed effect from the linear mixed-effects models for predicting each behavioral outcome measure was assessed at *α* = 0.05. 

### 3.2. Results

In the Pearson tests, significant correlations were found between MMN latency recorded in response to the vowel-change and percent correct phoneme detection (*r* = 0.53, *p* < 0.05) for /bu/ as well as percent correct sentence recognition (*r* = −0.40, *p* < 0.05) across the quiet and noise listening conditions. Significant correlations were also found between MMN amplitude recorded in response to the vowel-change and percent correct phoneme detection (*r* = −0.50, *p* < 0.05) and reaction time (*r* = 0.56, *p* < 0.01) for /bu/, as well as percent correct sentence recognition (*r* = −0.66, *p* < 0.01) across listening conditions. Similar trends were found between theta power in response to the vowel-change and percent correct phoneme detection (*r* = 0.41, *p* < 0.05) and behavioral reaction time (*r* = −0.49, *p* < 0.05) in response to the CV syllable /bu/, as well as behavioral sentence recognition (*r* = 0.59, *p* < 0.01) across listening conditions. Additionally, results revealed significant correlations between MMN latency recorded in response to the consonant-change and percent correct phoneme detection (*r* = −0.47, *p* < 0.05) for /da/ as well as sentence recognition (*r* = −0.53, *p* < 0.01) across the quiet and noise listening conditions (see Table 3 for a summary of correlation coefficients).

Repeated measures ANOVA results from Koerner et al. [57] showed significant effects of background noise on MMN latency (*F*(1, 14) = 29.43, *p* < 0.001), amplitude (*F*(1, 14) = 32.52, *p* < 0.001), and EEG theta power (*F*(1, 14) = 19.37, *p* < 0.001). Koerner et al. [57] also provided detailed results from the linear mixed-effects regression analysis (see replicated Table 4 for summary of regression model results). Linear mixed-effects models showed that both MMN latency (*F*(1, 40) = 7.86, *p* < 0.01) and spectral power in the theta band (*F*(1, 40) = 6.61, *p* < 0.05) were significant predictors of percent correct phoneme detection across listening conditions and stimuli. Additionally, MMN amplitude in response to the syllable /bu/ was a significant predictor of sentence recognition across listening conditions (*F*(1, 11) = 7.21, *p* < 0.05). As all residual plots from each linear mixed-effects model revealed that residuals were normally distributed without any signs of heteroscedastic variance or significant trends, we do not expect that generalized linear models would improve the results. Interactions were tested in previous models and were subsequently removed due to a lack of statistical significance.

## 4. Discussion

This current report compared results from Pearson correlations and linear mixed-effects regression models using data from two published ERP studies. It was determined that Pearson correlations were not appropriate for examining relationships in our data, which contained built-in differences across within-subject repeated measures. The results showed how linear mixed-effects regression models (after verification of normality of residuals and homogeneity of variance) are able to depict relationships between the predictor and outcome variables while taking into account repeated measures across participants. While the LME models were able to confirm basic conclusions gained from the Pearson correlation analyses for both studies [54,57], a comparison of methods and results for each model highlighted differences between the two approaches. 

The repeated measures ANOVA indicated that background noise had a significant effect on N1 and P2 latencies as well as N1 amplitudes in response to the syllable /bu/ [54]. Similarly, the repeated measures ANOVA revealed that MMN latency, amplitude, and spectral power were significantly impacted by background noise [57]. These results support the possibility that pooling data from quiet and noise listening conditions created a built-in contrast and bias between data points when Pearson correlations were used, which partly led to the overestimation of the association strength in the reported results (Table 1 and Table 3). In other words, the Pearson correlation analysis ignores these built-in differences and treats this type of data as if each variable in the repeated measures design were independent and normally distributed across the two listening conditions. The resulting *p*-values represent the probability of observing an effect that is as large, or larger, than what would be observed if there was no covariance structure in the repeated measures. In contrast, LME regression analysis was able to account for the covariance structure and grouping factors for the repeated measures. Tests of significance from the LME models examined whether each predictor variable, or fixed effect, was significantly different than zero while taking into account the other fixed or random effects in the model.

One issue common to regression analysis concerns the possible existence of multi-collinearity (or the existence of high correlations) among the predictor variables and how it may inflate the results with unstable estimates of regression coefficients such as an overall significant model with no significant predictors [2,3,4,5]. In the mixed-effects (or multilevel) models, the implementation of fixed and random effects allows control of the within-subject factor for repeated measures, and the additional stepwise approach allows removal of predictor variables in a systematic fashion, for instance, calculating a variance inflation factor (VIF) to identify collinear predictors to aid the stepwise removal of predictors from the LME models. The VIF represents the proportion of variance in one predictor variable accounted for by all the other predictors in the model. Estimation of VIFs for each predictor and progressive dropping of the predictor with the largest VIF beyond the cutoff criterion can be helpful in dealing with the collinearity of interaction terms. By contrast, Pearson correlation analysis assumes independence of the variables, and only fixed effects are directly examined piecewise without elaborate procedures to take into account how the existing associations/differences among the predictor variables may contribute to (oftentimes inflate) the correlation coefficients. The bivariate Pearson correlation analysis disregards potential correlations and data groupings among variables, which makes it inappropriate for research questions that aim to examine associations between variables that contain built-in differences between experimental conditions or subject groups.

Although the flexibility in model selection can be considered a strength of LME regression analysis, the number of educated choices a researcher must make while developing and implementing models can be a challenge. For instance, the inclusion of interactions or random effects in LME models affects the regression coefficients and interpretation of fixed effects, which cannot properly be taken into account in the bivariate Pearson correlation analysis. Although stepwise regression methods are available as a systematic approach to choose an appropriate model, it is important for researchers to think deeply about the subject matter in order to determine whether the inclusion and interpretation of specific fixed and random effects are appropriate for the specific research question and study objective.

While the two ERP studies reported here are clearly limited in scope and depth of analysis, the side-by-side comparisons clearly demonstrate the limitations and inappropriateness of the Pearson approach as well as its inflated correlation estimation results for the data sets. Given that multiple analysis techniques (for example, waveform analysis, source localization, time-frequency analysis) can be applied to the same neurophysiological data in cognitive neuroscience research [54,57,58,59], a cautionary note against the convenient use of the simple Pearson correlation test is necessary when selecting and applying statistical models to interpret brain-behavior correlations (e.g., biomarkers of various diseases and disorders) or correlations among the various brain measures with prior distributions and covariance structure for repeated measures. 

## 5. Conclusions

In sum, this report compared conventional Pearson correlations and linear mixed-effects (LME) regression models using data from two published auditory electrophysiology studies. The Pearson correlation test is inappropriate for the specific research questions in both studies as the neural responses across listening conditions were simply treated as independent measures. Although our comparative analysis is limited in its scope and depth, this technical note demonstrates the advantages as well as the necessity to apply mixed-effects models to properly account for the built-in relationships among the multiple predictor variables, which has important implications for proper modeling and interpretation of human behavior in terms of neural correlates and biomarkers. 

## Figures and Tables

**Table 1:** Correlation coefficients for relationship between-phase locking values and N1 and P2 latency and amplitude values in response to the CV syllable /bu/ at electrode Cz as reported in Koerner and Zhang [54].

	N1		P2	
Frequency Band	Latency	Amplitude	Latency	Amplitude
Delta	−0.586 **	−0.780 ***	−0.468 *	0.666 **
Theta	−0.521 *	−0.765 ***	−0.575 **	0.612 **
Alpha	−0.510 *	−0.720 ***	−0.586 **	0.599 **

*** *p* < 0.001; ** *p* < 0.01; * *p* < 0.05.

**Table 2:** F-statistics and regression coefficients (β) for each fixed effect from linear mixed-effects regression models for N1–P2 latencies and amplitudes.

Variable	N1 Latency		N1 Amplitude		P2 Latency		P2 Amplitude	
	F	β	F	β	F	β	F	β
Intercept	964.79 ***	-	155.62 ***	-	568.62 ***	-	31.64 ***	-
Condition	106.88 ***	-	16.58 **	-	31.93 ***	-	4.13	-
Delta	0.06	−0.30	16.12 **	−1.05	0.46	0.48	10.72 *	0.96
Theta	0.46	−0.45	0.17	−1.82	4.01	−0.23	0.00	−0.11
Alpha	12.51 **	0.80	3.24	2.01	0.68	−0.41	0.00	0.09

*** *p* < 0.001; ** *p* < 0.01; * *p* < 0.05.

**Table 3:** Correlation coefficients for brain-behavior correlations between neural MMN latency, amplitude, and theta power for /bu/ and /da/ at electrode Cz and behavioral phoneme detection percent correct, reaction time, and percent correct sentence recognition scores.

	Latency (ms)		Amplitude (μV)		Power (dB)	
	/bu/	/da/	/bu/	/da/	/bu/	/da/
Phoneme Detection (%)	−0.53 *	−0.47 *	−0.50 *	−0.17	0.41 *	0.13
Reaction Time (ms)	0.34	0.39	0.56 **	0.02	−0.49 *	0.01
Sentence Recognition (%)	−0.40 *	−0.53 **	−0.66 **	−0.07	0.59 **	0.18

*** *p* < 0.001; ** *p* < 0.01; * *p* < 0.05.

**Table 4:** F-statistics and regression coefficients (β) for fixed effects from linear mixed-effects regression models for each behavioral measure (Koerner et al. [57]).

Variable	Percent Correct Phoneme Detection		Phoneme Detection Reaction Time		Percent Correct Sentence Recognition (/bu/)		Percent Correct Sentence Recognition (/da/)	
	F	β	F	β	F	β	F	β
Intercept	161.51 ***	-	4199.98 ***	-	431.41 ***	-	335.12 ***	-
Condition	131.68 ***	-	61.92 ***	-	291.32 ***	-	247.69 ***	-
Stimulus	114.20 ***	-	21.05 ***	-	-	-	-	-
Latency	7.86 **	0.61	0.000	0.03	1.24	−0.19	0.44	−0.21
Amplitude	3.10	−0.09	0.002	0.02	7.21 *	0.24	0.41	0.05
Theta Power	6.61 *	0.05	0.368	0.01	0.46	−0.01	1.50	−0.02

*** *p* < 0.001; ** *p* < 0.01; * *p* < 0.05.

## Abbreviations

The following abbreviations are used in this manuscript:

LME	Linear mixed-effects
GLME	Generalized linear mixed-effects
EEG	Electroencephalography
ERP	Event related potential
SNR	Signal-to-noise ratio
N1	Negative-going ERP response that peaks at approximately 100 ms after auditory stimulus onset
P2	Positive-going ERP response that follows the N1
ANOVA	Analysis of variance
nlme	A R package for linear and nonlinear mixed effects models
CV	Syllable structure consisting of a consonant and vowel (e.g., /ba/)
F	F statistic, a value from ANOVA or regression analysis to indicate differences between two means
β	Vector of fixed effect slopes in the linear mixed effects models
μV	Microvolt
dB	Decibel
VIF	Variance inflation factor
